# TuberOus SClerosis registry to increAse disease awareness (TOSCA) Post-Authorisation Safety Study of Everolimus in Patients With Tuberous Sclerosis Complex

**DOI:** 10.3389/fneur.2021.630378

**Published:** 2021-03-23

**Authors:** J. Chris Kingswood, Elena Belousova, Mirjana P. Benedik, Klemens Budde, Tom Carter, Vincent Cottin, Paolo Curatolo, Maria Dahlin, Lisa D'Amato, Guillaume B. d'Augères, Petrus J. de Vries, José C. Ferreira, Martha Feucht, Carla Fladrowski, Christoph Hertzberg, Sergiusz Jozwiak, John A. Lawson, Alfons Macaya, Ruben Marques, Rima Nabbout, Finbar O'Callaghan, Jiong Qin, Valentin Sander, Matthias Sauter, Seema Shah, Yukitoshi Takahashi, Renaud Touraine, Sotiris Youroukos, Bernard Zonnenberg, Anna C. Jansen, TOSCA Consortium and TOSCA Investigators

**Affiliations:** ^1^Genomics Clinical Academic Group, Molecular and Clinical Sciences Research Centre, St George's Hospital, University of London, London, United Kingdom; ^2^Research and Clinical Institute of Paediatrics, Pirogov Russian National Research Medical University, Moscow, Russia; ^3^SPS Pediatrična Klinika, Ljubljana, Slovenia; ^4^Internal Medicine and Nephrology, Hypertensiology DHL, University Medicine Berlin, Berline, Germany; ^5^Tuberous Sclerosis Association, Nottingham, United Kingdom; ^6^Hôpital Louis Pradel, Claude Bernard University Lyon, Lyon, France; ^7^Tor Vergata University Hospital, Rome, Italy; ^8^Karolinska University Hospital, Stockholm, Sweden; ^9^Novartis Farma S.p.A., Origgio, Italy; ^10^Association Sclérose Tubéreuse de Bourneville, Gradignan, France; ^11^Division of Child and Adolescent Psychiatry, University of Cape Town, Cape Town, South Africa; ^12^Centro Hospitalar Lisboa Ocidental, Lisbon, Portugal; ^13^Universitätsklinik für Kinder-und Jugendheilkunde, Vienna, Austria; ^14^Associazione Sclerosi Tuberosa ONLUS, Milan, Italy; ^15^European Tuberous Sclerosis Complex Association, In den Birken, Dattein, Neuharlingersiel, Germany; ^16^Vivantes-Klinikum Neukölln, Berlin, Germany; ^17^Department of Child Neurology, Medical University of Warsaw, Warsaw, Poland; ^18^Department of Neurology and Epileptology, The Children's Memorial Health Institute, Warsaw, Poland; ^19^The Tuberous Sclerosis Multidisciplinary Management Clinic, Sydney Children's Hospital, Randwick, NSW, Australia; ^20^Hospital Universitari Vall d'Hebron, Barcelona, Spain; ^21^Institute of Biomedicine (IBIOMED), University of León, León, Spain; ^22^Department of Paediatric Neurology, Necker Enfants Malades Hospital, Paris Descartes University, Paris, France; ^23^Institute of Child Health, University College London, London, United Kingdom; ^24^Department of Paediatrics, Peking University People's Hospital, Beijing, China; ^25^Tallinn Children Hospital, Tallinn, Estonia; ^26^Klinikverbund Kempten-Oberallgäu gGmbH, Kempten, Germany; ^27^Novartis Healthcare Pvt. Ltd, Hyderabad, India; ^28^National Epilepsy Center, Shizuoka Institute of Epilepsy and Neurological Disorders, NHO, Shizuoka, Japan; ^29^Department of Genetics, CHU-Hôpital Nord, Saint Etienne, France; ^30^St. Sophia Children's Hospital, Athens, Greece; ^31^University Medical Center, Utrecht, Netherlands; ^32^Pediatric Neurology Unit, Department of Paediatrics, UZ Brussel VUB, Brussels, Belgium

**Keywords:** everolimus, TOSCA, tuberous sclerosis complex, post-authorization safety study, mammalian target of rapamycin

## Abstract

This non-interventional post-authorisation safety study (PASS) assessed the long-term safety of everolimus in patients with tuberous sclerosis complex (TSC) who participated in the TuberOus SClerosis registry to increase disease Awareness (TOSCA) clinical study and received everolimus for the licensed indications in the European Union. The rate of adverse events (AEs), AEs that led to dose adjustments or treatment discontinuation, AEs of potential clinical interest, treatment-related AEs (TRAEs), serious AEs (SAEs), and deaths were documented. One hundred seventy-nine patients were included in the first 5 years of observation; 118 of 179 patients had an AE of any grade, with the most common AEs being stomatitis (7.8%) and headache (7.3%). AEs caused dose adjustments in 56 patients (31.3%) and treatment discontinuation in nine patients (5%). AEs appeared to be more frequent and severe in children. On Tanner staging, all patients displayed signs of age-appropriate sexual maturation. Twenty-two of 106 female (20.8%) patients had menstrual cycle disorders. The most frequent TRAEs were stomatitis (6.7%) and aphthous mouth ulcer (5.6%). SAEs were reported in 54 patients (30.2%); the most frequent SAE was pneumonia (>3% patients; grade 2, 1.1%, and grade 3, 2.8%). Three deaths were reported, all in patients who had discontinued everolimus for more than 28 days, and none were thought to be related to everolimus according to the treating physicians. The PASS sub-study reflects the safety and tolerability of everolimus in the management of TSC in real-world routine clinical practice.

## Introduction

Tuberous sclerosis complex (TSC) is a rare, genetic, multisystem disorder. TSC can affect almost any organ system, including the skin, central nervous system, kidneys, eye, heart, and lung. About 90% of the TSC patients experience neurological and renal abnormalities, which represent a major cause of morbidity and mortality (1, 2). The clinical presentation of TSC is heterogeneous, and the degree of severity is highly variable between individuals, even among the family members (1). The onset of clinical manifestations of TSC also typically varies with age, which further adds to the complexity to the disease (3, 4). These factors represent a significant challenge for the diagnosis and management of TSC. Current management guidelines are focused on early identification and close monitoring of lesion burden in combination with timely medical treatment of manifestations and early interventions for TSC-associated neuropsychiatric disorders (TANDs) (4). TSC is caused by pathogenic variants in either *TSC1* or *TSC2* genes, resulting in hyper-activation of the mammalian target of rapamycin (mTOR) signalling pathway and the subsequent development of hamartomatous lesions in patients with TSC (4).

Based on double-blinded, placebo-controlled, randomised, clinical trials that confirmed its safety and efficacy, the mTOR-inhibitor everolimus (Votubia®) was approved in Europe in 2011 for the treatment of subependymal giant cell astrocytoma (SEGA) and renal angiomyolipoma (5–13). Randomised clinical trial studies are required as “gold standard” for product licensing. However, they fail to reflect the “real-world” scenarios, particularly in terms of AE representation. The randomised clinical trials have shown that everolimus was generally well-tolerated in patients with TSC with manageable AEs, which were generally reversible and non-cumulative (14–16). However, since TSC is a rare disease, with a prevalence of 6.8–12.4 per 100,000 people (17), the three key registration trials included relatively small numbers of patients with TSC [ranging from 78 in EXIST-1 to 247 in EXIST-3 (5, 9, 12)].

The TuberOus SClerosis registry to increase disease Awareness (TOSCA) study was conducted to address existing lacunas in the diagnosis and management of TSC. Based on the request from European Medicines Agency (EMA) to use the TOSCA registry to collect data on long-term safety and reproductive abnormalities in patients taking everolimus for licensed indications, SEGA in children age 2–20 years, and angiomyolipoma in adults aged >18 years, the TOSCA post-authorisation safety study (PASS) was developed. Here, we report findings from this TOSCA sub-study.

## Methods

### Study Design and Participants and Data Collection

The TOSCA clinical study methodology has been published previously (15). In brief, TOSCA was a large-scale non-interventional study in patients with TSC. The study was designed with a core section, six ancillary research projects (with more detailed focus on SEGA, renal angiomyolipoma and lymphangiomyomatosis, genetics, TAND, epilepsy, and patient's quality of life), and a PASS sub-study (EU PASS Register Number EUPAS3247).

The TOSCA-PASS sub-study was aimed at collecting prospective long-term safety data of treatment with everolimus prescribed for the indications licensed in Europe at time of enrolment data on AEs, therapeutic drug monitoring data, and the long-term reproductive abnormalities within routine clinical practice were collected. The PASS sub-study was conducted in 11 European Union countries participating in the TOSCA registry.

### Patients

Patients who participated in the TOSCA registry and received everolimus treatment in the licensed indications (for SEGA or renal angiomyolipoma) in the European Union were eligible for inclusion in the TOSCA PASS, after providing additional written informed consent.

The data collection cut-off was 10 August 2017 for the TOSCA PASS sub-study. As per EMA indication (EMA/CHMP/59467/2014, 20 February 2014), data collection on sexual maturation and fertility is to be continued for all paediatric patients until they reach Tanner stage 5, or age 16 years for females and age 17 years for males, whichever occurs first.

For the TOSCA PASS sub-study, being a non-interventional and observational study, all treatment-related decisions (dose adjustments, treatment discontinuation) were at the discretion of the treating physicians. No treatment protocol, diagnostic/therapeutic procedure, or a formal visit schedule was mandated by the TOSCA PASS study protocol. However, the recommended data collection per study schedule was at 3-monthly intervals, which most likely mirrors the patterns of routine clinical care of patients treated with everolimus. Detailed management of each individual's AEs was not collected; however, general guidelines were followed by investigators (18). Data were collected for all patients who achieved Tanner stage 5 by the cut-off date. For patients who discontinued the study prematurely, the reason for discontinuation was determined. All patients were instructed regarding possible AEs and their possible treatment (19).

In this manuscript, we present an interim analysis of patient data up to 10 August 2017. The long-term safety data of these patients will be reported after termination of the TOSCA PASS study.

### Outcome Measures and Data Analysis

Incidence of AEs, AEs that lead to dose adjustment or discontinuation, everolimus treatment-related AEs (TRAEs) as per the investigator assessment, AEs of special interest (AESIs), serious AEs (SAEs), and deaths were documented during the treatment (from day of enrolling into the PASS study to 28 days after the last dose of everolimus). AESIs were those AEs that were of specific clinical interest in connection with everolimus treatment. Potential AEs sought included non-infectious pneumonitis, severe infections, hypersensitivity (anaphylactic reactions), stomatitis, wound healing complications, increased serum creatinine/proteinuria/renal failure, hyperglycaemia, new onset of diabetes mellitus, dyslipidaemia, hypophosphatemia, cardiac failure, cytopenias, haemorrhages, thrombotic and embolic events, female fertility (including secondary amenorrhea), pre-existing infections (reactivation/aggravation/exacerbation), safety in patients with hepatic impairment, postnatal developmental toxicity, pregnant or breast-feeding women, male infertility, and muscle wasting/muscle loss. The relationship of the incidence of AESIs with everolimus blood levels was also noted. SAEs were defined as AEs that are fatal or life-threatening, result in persistent or significant disability/incapacity, constitute a congenital anomaly/birth defect, are medically significant, require medical or surgical intervention, or require inpatient hospitalisation or prolonged existing hospitalisation.

Data on everolimus (dose, interruption, dose change, and duration) and concomitant medication were captured. Concomitant medications entered into the database were coded using the World Health Organization (WHO) drug reference list, which employs the anatomical therapeutic chemical (ATC) classification system.

The analysis set consisted of all patients who had at least one post-baseline safety assessment and were exposed to at least one dose of everolimus after the enrolment. The AEs and SAEs were summarised by system organ class and preferred term using the Medical Dictionary for Regulatory Activities (MedDRA) version 20.

## Results

### Patient Disposition and Baseline Characteristics

**Figure 1:**
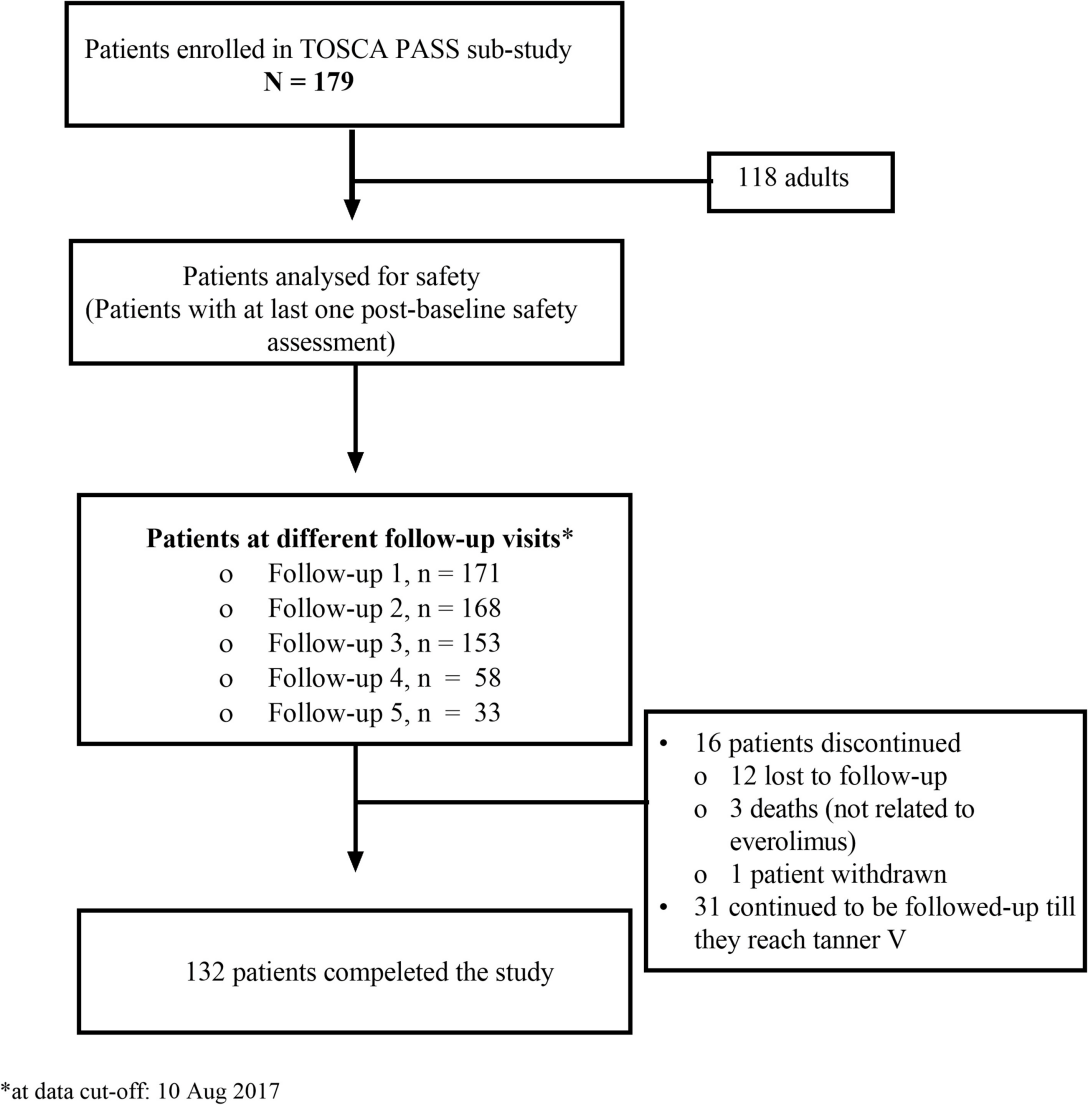
Patient disposition in TOSCA PASS sub-study. TOSCA, TuberOus SClerosis registry to increase disease Awareness; PASS, post-authorisation safety study.

A total of 179 patients were enrolled in the study. Of 179 patients, 16 patients (8.9%) had discontinued participation in the study, and 31 patients continued to be followed up as part of the ongoing paediatric PASS. Of the 16 patients who discontinued the study, 12 were lost to follow-up, three died, and one patient was withdrawn as per investigator's decision (Figure 1). Everolimus was initiated for 73 (40.8%) patients with SEGA, 122 (68.2%) with renal angiomyolipoma, and 17 (9.5%) for both SEGA and renal angiomyolipoma.

The demographic and clinical characteristics of the enrolled patients are summarised in Table 1. Of 179 patients enrolled, 61 (34.1%) were children (<18 years), and 73 and 106 (59.2%) were female. The median age at consent was 27 years (range < 1–65 years). So far, all patients have reached their 1-year follow-up visit; while 175, 158, 75, and 37 patients have completed their second, third, fourth, and fifth annual follow-up visits, respectively. Mutation testing was performed on 92 patients (51.4%). The majority of patients, 71 (77.2%), had pathogenic variants in *TSC2*, 8 (8.7%) had pathogenic variants in *TSC1*, and 13 (14.1%) remained without genetic diagnosis (no mutation identified or NMI).

**Table 1:** Demographic and clinical characteristics of the enrolled patients at baseline.

**Characteristics**	**Patients**
	***N* = 179**
**Sex, n (%)**	
Male	73 (40.8)
Female	106 (59.2)
**Age at consent, years**	
Mean (SD)	27.1 (16.08)
Median (range)	27.0 (<1–65)
**Age groups, n (%)**	
≤2 years	7 (3.9)
>2 to ≤5 years	6 (3.4)
>5 to ≤9 years	21 (11.7)
>9 to ≤14 years	16 (8.9)
>14 to ≤18 years	11 (6.1)
≥18 to ≤40 years	77 (43.0)
>40 years	41 (22.9)
**Geographic region**	
Netherlands	75 (41.9)
Germany	43 (24.0)
France	19 (10.6)
Spain	17 (9.5)
Austria	12 (6.7)
Czech Republic	3 (1.7)
Slovenia	3 (1.7)
United Kingdom	3 (1.7)
Sweden	2 (1.1)
Denmark	1 (0.6)
Poland	1 (0.6)
**Patients with molecular testing, n (%)**	92 (51.4)
*TSC1* mutation	8 (8.7)
*TSC2* mutation	71 (77.2)
No mutation identified	13 (14.1)
**TSC manifestations, n (%)**	
Neurological	
SEGA	100 (55.9)
Cortical tuber	147 (82.1)
SEN	156 (87.2)
Cerebral white matter radial migration lines	24 (13.4)
Renal	
Renal angiomyolipoma	149 (83.2)
Multiple renal cysts	53 (29.6)
Polycystic kidneys	4 (2.2)
Impaired renal function	5 (2.8)
Renal malignancy	1 (0.6)
Pulmonary	
Lymphangioleiomyomatosis	32 (17.9)
Cardiovascular	
Cardiac rhabdomyoma	51 (28.5)
Dermatologic	
≥3 hypomelanotic macules	84 (46.9)
Facial angiofibroma	123 (68.7)
Shagreen patch	42 (23.5)
Ungual or periungual fibromas	44 (24.6)
Forehead plaque	19 (10.6)
Confetti lesions	16 (8.9)
Ophthalmologic	
Retinal hamartoma	32 (17.9)
Epilepsy	151 (84.4)

*SD, standard deviation; SEGA, subependymal giant cell astrocytoma; SEN, subependymal nodule; TSC, tuberous sclerosis complex*.

### Safety

Overall, 118 of 179 patients (65.9%) had AEs of any grade, irrespective of its relationship with study drug (Table 2). The most common grade 3/4 AE that occurred in >3% of patients was pneumonia observed in 2.8% of patients (Table 3). The rate of AEs was higher in children compared with adults [75.4% (*n* = 61) vs. 61.0% (*n* = 72); *P* = 0.0541], and a decreasing trend on AE rate was noted with increase in age (Table 2).

**Table 2:** Overall AE profile in overall population and across age groups.

	**Overall (*N* = 179) n (%)**	**Age at consent, years**				
		**≤2 (*N* = 7) n (%)**	**>2 to ≤9 (*N* = 27) n (%)**	**>9 to <18 (*N* = 27) n (%)**	**≥18 (*N* = 118) n (%)**	***P*-value**
Overall, any AEs	118 (65.9)	6 (85.7)	21 (77.8)	19 (70.4)	72 (61)	
Patients with frequent (>3%) adverse events with CTC grade						
Grade 1	40 (22.3)	2 (28.6)	8 (29.6)	8 (29.6)	22 (18.6)	
Grade 2	27 (15.1)	1 (14.3)	2 (7.4)	4 (14.8)	20 (16.9)	
Grade 3	16 (8.9)	2 (28.6)	5 (18.5)	2 (7.4)	7 (5.9)	
Grade 4	2 (1.1)	0	0	0	2 (1.7)	
Patients with grade 3/4 AEs	43 (24.0)	4 (57.1)	8 (29.6)	6 (22.2)	25 (21.2)	
Patients with SAE	54 (30.2)	5 (71.4)	12 (44.4)	7 (25.9)	30 (25.4)	0.0567
AE requiring dose adjustment	56 (31.3)	5 (71.4)	10 (37.0)	9 (33.3)	32 (27.1)	0.1342
AE leading to discontinuation	9 (5.0)	1 (14.3)	1 (3.7)	1 (3.7)	6 (5.1)	0.8357
Treatment-related AE	76 (42.5)	6 (85.7)	15 (55.6)	16 (59.3)	39 (33.1)	0.0023
Deaths	3 (1.7)	0	0	0	3 (2.5)	

*AE, adverse event; CTC, Common Terminology Criteria; SAE, serious adverse events*.

**Table 3:** Adverse events of any cause (by preferred term) reported in >3% of patients in overall population and across age groups.

**Adverse events**	**Overall** ***N*** **= 179**		**Age at consent, years**							
			**≤2**		**>2 to ≤9**		**>9 to <18**		**≥18**	
			**(*****N*** **= 7)**		**(*****N*** **= 27)**		**(*****N*** **= 27)**		**(*****N*** **= 118)**	
	**All**	**Grade 3/4**	**All**	**Grade 3/4**	**All**	**Grade 3/4**	**All**	**Grade 3/4**	**All**	**Grade 3/4**
	**n (%)**	**n (%)**	**n (%)**	**n (%)**	**n (%)**	**n (%)**	**n (%)**	**n (%)**	**n (%)**	**n (%)**
Stomatitis	14 (7.8)	1 (0.6)	1 (14.3)	0	3 (11.1)	1 (3.7)	5 (18.5)	0	5 (4.2)	0
Headache	13 (7.3)	0	0	0	0	0	3 (11.1)	0	10 (8.5)	0
Diarrhoea	12 (6.7)	1 (0.6)	1 (14.3)	0	3 (11.1)	0	1 (3.7)	0	7 (5.9)	1 (0.8)
Vitamin D deficiency	12 (6.7)	0	0	0	0	0	0	0	12 (10.2)	0
Aphthous ulcer	10 (5.6)	0	0	0	2 (7.4)	0	1 (3.7)	0	7 (5.9)	0
Hypercholesterolaemia	10 (5.6)	0	2 (28.6)	0	3 (11.1)	0	2 (7.4)	0	3 (2.5)	0
Urinary tract infection	10 (5.6)	1 (0.6)	1 (14.3)	1 (14.3)	0	0	1 (3.7)	0	8 (6.8)	0
Pyrexia	9 (5.0)	0	3 (42.9)	0	3 (11.1)	0	2 (7.4)	0	1 (0.8)	0
Hypertension	8 (4.5)	2 (1.1)	0	0	0	0	1 (3.7)	0	7 (5.9)	2 (1.7)
Pneumonia	8 (4.5)	5 (2.8)	2 (28.6)	1 (14.3)	2 (7.4)	2 (7.4)	1 (3.7)	0	3 (2.5)	2 (1.7)
Viral upper respiratory tract infection	8 (4.5)	0	0	0	1 (3.7)	0	1 (3.7)	0	6 (5.1)	0
Abdominal pain	7 (3.9)	1 (0.6)	0	0	2 (7.4)	1 (3.7)	0	0	5 (4.2)	0
Anaemia	7 (3.9)	1 (0.6)	0	0	1 (3.7)	0	1 (3.7)	0	5 (4.2)	1 (0.8)
Bronchitis	7 (3.9)	0	2 (28.6)	0	3 (11.1)	0	1 (3.7)	0	1 (0.8)	0
Oedema peripheral	7 (3.9)	0	0	0	1 (3.7)	0	0	0	6 (5.1)	0
Epilepsy	6 (3.4)	2 (1.1)	1 (14.3)	1 (14.3)	1 (3.7)	0	1 (3.7)	0	3 (2.5)	1 (0.8)
Hypertriglyceridaemia	6 (3.4)	2 (1.1)	0	0	0	0	4 (14.8)	2 (7.4)	2 (1.7)	0
Influenza	6 (3.4)	3 (1.7)	1 (14.3)	1 (14.3)	1 (3.7)	0	0	0	4 (3.4)	2 (1.7)
Vomiting	6 (3.4)	1 (0.6)	1 (14.3)	0	3 (11.1)	1 (3.7)	0	0	2 (1.7)	0

The most frequent TRAEs were stomatitis (6.7%), aphthous mouth ulcer (5.6%), and hypercholesterolaemia (5%). Everolimus dose adjustments due to AEs were reported in 56 patients (31.3%). The most common AEs that led to dose adjustments in at least two patients were diarrhoea (five patients); stomatitis, pneumonia, common cold, and urinary tract infection (three patients each); and metrorrhagia, pyrexia, pyelonephritis, sinusitis, influenza, and otitis (two patients each). Haemorrhagic events leading to dose adjustments were haemorrhage on the left side of the brain, bleeding in angiomyolipoma, and renal haemorrhage (one patient each). AEs leading to everolimus discontinuation were reported in nine patients (5%) and included fatigue and amenorrhea (1.1% each); and anaemia, mouth ulceration, empyema, pneumonia, hyperglycaemia, type I diabetes mellitus, flank pain, intestinal adenocarcinoma, seizure, and alopecia (0.6% each).

SAEs were reported in 54 patients (30.2%). The most frequent SAE (>3% of patients) was pneumonia (grade 2, 1.1%; grade 3, 2.8%).

Three deaths were reported in the study. These deaths occurred after 28 days of everolimus discontinuation and were reported by study investigators as not related to everolimus treatment. Patient 1, male, aged 30 years, from the Netherland, died due to medically assisted death as per the local regulations on day 487 after commencement of everolimus administration. He had permanently discontinued everolimus treatment on day 205. Patient 2, male aged 52 years, died from influenza (grade 4) on day 1399; everolimus treatment was permanently discontinued on day 1359. Patient 3, male aged 46 years, died due to intestinal adenocarcinoma on day 74; everolimus was permanently discontinued on day 43. Autopsy was not performed for patients 1 and 2, whereas, this was unknown for patient 3.

### Everolimus Dosage and Exposure

Data on everolimus dosage and exposure were available for 150 patients. The mean duration of the everolimus exposure was 302.4 ± 105.04 days (median, 365 days; range, 7–669 days). The mean and median daily doses and the most commonly administered dosage (5 mg throughout the study) are shown in Table 4. The mean everolimus blood level at baseline was 6.27 ng/ml (median, 4.9 ng/ml; range, 1.4–35.9 ng/ml), with the maximum concentration reported at third follow-up visit (mean, 6.6 ng/ml) and the least at fourth follow-up visit (mean 4.937 ng/ml). Median duration of exposure in <18 years (*n* = 59) and ≥18 (*n* = 91) was 365 days (*P* = 0.1735) with a significant difference in mean daily everolimus dose of 6.4 mg (range, 1–13 mg) in <18 years vs. 7.7 mg (range, 1–20 mg) in ≥18 years (*P* = 0.0144), respectively. Changes in everolimus dosage were reported in 53 patients (35.3%), with dose increase reported in 44 patients (83%), dose interruptions in 20 patients (13.3%), and dose reductions in 14 patients (9.3%) (Table 4). The most common reasons for dose changes were side effects (21 patients, 14%); other reasons are specified in Table 4.

**Table 4:** Everolimus dosage and exposure.

	**Baseline**	**FU1**	**FU2**	**FU3**	**FU4**	**FU5**
	**(*N* = 150)**	**(*N* = 171)**	**(*N* = 168)**	**(*N* = 153)**	**(*N* = 58)**	**(*N* = 33)**
**Pharmaceutical formulation**						
Tablets	143 (95.3)	165 (96.5)	162 (96.4)	147 (96.1)	56 (96.6)	33 (100.0)
Dispersible tablets	9 (6.0)	9 (5.3)	8 (4.8)	10 (6.5)	3 (5.2)	0
**Dosage**						
2 mg	3 (2.0)	3 (1.8)	3 (1.8)	3 (2.0)	0	0
2.5 mg	16 (10.7)	13 (7.6)	19 (11.3)	17 (11.1)	6 (10.3)	2 (6.1)
3 mg	2 (1.3)	3 (1.8)	2 (1.2)	2 (1.3)	1 (1.7)	0
5 mg	118 (78.7)	131 (76.6)	128 (76.2)	114 (74.5)	43 (74.1)	31 (93.9)
10 mg	17 (11.3)	18 (10.5)	16 (9.5)	16 (10.5)	4 (6.9)	0
Other	34 (22.7)	37 (21.6)	25 (14.9)	18 (11.8)	6 (10.3)	0
**Daily dose (mg)**						
Mean (SD)	7.2 (3.11)	7.3 (3.14)	7.1 (3.28)	7.4 (4.27)	7.8 (3.40)	8.3 (3.99)
Median (min–max)	7.0 (1–20)	7.5 (1–20)	6.4 (0–20)	5.8 (0–35)	7.5 (3–15)	7.5 (3–15)
**Patients with dose changes**	53 (35.3)	55 (32.2)	52 (31.0)	34 (22.2)	19 (32.8)	0
Reductions	14 (9.3)	15 (8.8)	24 (14.3)	9 (5.9)	3 (5.2)	0
Interruptions	20 (13.3)	31 (18.1)	26 (15.5)	22 (14.4)	10 (17.2)	0
Increased	44 (29.3)	41 (24.0)	34 (20.2)	23 (15.0)	17 (29.3)	0
**Reasons for changes**						
Side effect	21 (14.0)	25 (14.6)	22 (13.1)	11 (7.2)	2 (3.4)	0
Dosing error	3 (2.0)	1 (0.6)	1 (0.6)	0	0	0
Lab test abnormality	2 (1.3)	4 (2.3)	1 (0.6)	0	0	0
Concomitant medication affecting drug exposure	1 (0.7)	2 (1.2)	0	0	0	0
Other	30 (20.0)	30 (17.5)	24 (14.3)	20 (13.1)	10 (17.2)	0

*FUP, follow-up; SD, standard deviation*.

### Correlations Between Everolimus Blood Levels and Adverse Events of Special Interest

AESIs were reported in 57 of 150 patients (38%) for whom data on everolimus dosage and exposure are available. AESIs were suspected to be everolimus-related in 40 patients (26.7%). The majority of the patients had grade 1 or grade 2 AEs. One patient reported grade 4 empyema. Most of the patients who experienced AESIs had everolimus concentration <8 ng/ml (24%). No significant correlation was observed between everolimus blood concentration and AESIs (Table 5).

**Table 5:** Correlation between everolimus exposure and incidence of AESIs at baseline.

**Time from baseline visit**	**Patients with AESI**	**Everolimus concentration (ng/ml), n (%)**					***P*-value**
		**<3**	**3 to <7**	**7 to <9**	**9 to ≤15**	**>15**	
Quarter 1 (*n* = 57)	Yes	4 (7)	10 (17.5)	0	1 (1.8)	0	0.1673
	No	12 (21.1)	15 (26.3)	7 (12.3)	4 (7)	4 (7)	
Quarter 2 (*n* = 59)	Yes	4 (6.8)	11 (18.6)	3 (5.1)	2 (3.4)	0	0.7195
	No	10 (16.9)	19 (32.2)	3 (5.1)	5 (8.5)	2 (3.4)	
Quarter 3 (*n* = 71)	Yes	5 (7)	9 (12.7)	0	5 (7)	1 (1.4)	0.2557
	No	8 (11.3)	24 (33.8)	9 (12.7)	7 (9.9)	3 (4.2)	
Quarter 4 (*n* = 67)	Yes	4 (6)	8 (11.9)	3 (4.5)	4 (6)	0	0.4535
	No	7 (10.4)	24 (35.8)	7 (10.4)	5 (7.5)	5 (7.5)	

*Quarters 1 to 4 denote the quarter of year at the baseline visit. An event is mapped into Q j of Baseline if its start date is prior to Baseline date + 3 multiply by j months/Baseline date + (3 multiply by j + 12) months and {its stop date is on or after the Baseline date + 3 multiply by (j – 1) months/Baseline date + [3 multiply by (j – 1) + 12] months or the event is ongoing}, where j = 1, 2, 3, 4*.

*AESI, adverse event of special interest*.

### Sexual Maturation and Menstrual Irregularities

Tanner staging was performed in 28 patients (15.6%; three male and 25 females). There were no significant delays in sexual maturation revealed (Table 6). Nineteen females (17.9%) used contraception, with the most commonly contraception being hormone-based contraception in 16 patients (84.2%). Overall, three patients (1.7%) had ovariectomy, and five (2.8%) used external sex hormones (Table 6).

**Table 6:** Sexual maturation and menstrual irregularities across age groups.

		**Age at consent, years**						
	**Overall (*N* = 179)**	**≤2 (*N* = 7)**	**2 to ≤5 (*N* = 6)**	**5 to ≤9 (*N* = 21)**	**>9 to ≤14 (*N* = 16)**	**>14 to ≤18 (*N* = 11)**	**>18 to ≤40 (*N* = 77)**	**>40 (*N* = 41)**
Total patients evaluated for Tanner stages	28 (15.6)	0	1 (6.7)	4 (19.0)	10 (62.5)	6 (54.5)	6 (7.8)	1 (2.4)
Male patients with Tanner stages evaluated	3 (10.7)	0	0	2 (50.0)	1 (10.0)	0	0	0
Male patients with genitalia stage	3 (4.1)	0	0	2 (14.3)	1 (20.0)	0	0	0
Stage 1	0	0	0	0	0	0	0	0
Stage 2	0	0	0	1 (50.0)	0	0	0	0
Stage 3	1 (33.3)	0	0	0	1 (100.0)	0	0	0
Stage 4	1 (33.3)	0	0	1 (50.0)	0	0	0	0
Stage 5	1 (33.3)	0	0	0	0	0	0	0
Patients with pubic hair stage	3 (4.1)	0	0	2 (14.3)	1 (20.0)	0	0	0
Stage 1	1 (33.3)	0	0	0	0	0	0	0
Stage 2	1 (33.3)	0	0	1 (50.0)	0	0	0	0
Stage 3	2 (66.7)	0	0	0	1 (100.0)	0	0	0
Stage 4	0	0	0	0	0	0	0	0
Stage 5	1 (33.3)	0	0	1 (50.0)				
Female patients with Tanner stages evaluated	25 (89.3)	0	1 (16.7)	2 (50.0)	9 (90.0)	6 (100.0)	6 (100.0)	1 (100.0)
Patients with breast stage	23 (21.7)	0	1 (33.3)	2 (28.6)	8 (72.7)	7 (70.0)	5 (10.2)	1 (4.0)
Stage 1	2 (8.7)	0	1 (100.0)	1 (50.0)	0	0	0	0
Stage 2	1 (4.3)	0	0	0	0	1 (16.7)	0	0
Stage 3	3 (13.0)	0	0	0	3 (37.5)	0	0	0
Stage 4	3 (13.0)	0	0	1 (50.0)	2 (25.0)	0	0	0
Stage 5	14 (60.9)	0	0	0	3 (37.5)	5 (83.3)	5 (100.0)	1 (100.0)
Patients with pubic hair stage	24 (22.6)	0	1 (33.3)	2 (28.6)	9 (81.8)	7 (70.0)	5 (10.2)	1 (4.0)
Stage 1	2 (8.3)	0	0	1 (50.0)	0	1 (16.7)	0	0
Stage 2	2 (8.3)	0	0	0	2 (22.2)	0	0	0
Stage 3	3 (12.5)	0	1 (100.0)	0	2 (22.2)	0	0	0
Stage 4	1 (4.2)	0	0	0	1 (11.1)	0	0	0
Stage 5	16 (66.7)	0	0	1 (50.0)	4 (44.4)	5 (83.3)	5 (100.0)	1 (100.0)
Contraception use								
Patients who used contraception	19 (17.9)	0	0	1 (14.3)	0	2 (25.0)	12 (24.5)	4 (16.0)
Patients with hormone-based contraception	16 (84.2)	0	0	1 (100.0)	0	2 (100.0)	10 (83.3)	3 (75.0)
Type of hormone-based contraception								
Ethinyl oestradiol/progestin combination								
Overall	8 (50.0)	0	0	0	0	1 (50.0)	5 (50.0)	2 (66.7)
>50 μg of ethinyl oestradiol	3 (37.5)	0	0	0	0	0	2 (40.0)	1 (50.0)
<50 μg of ethinyl oestradiol	5 (62.5)	0	0	0	0	1 (100.0)	3 (60.0)	1 (50.0)
Progestin only								
Overall	8 (50.0)	0	0	1 (100.0)	0	1 (50.0)	5 (50.0)	1 (33.3)
Pill	4 (50.0)	0	0	1 (100.0)	0	0	3 (60.0)	0
Intrauterine devices	0	0	0	0	0	0	0	0
Depot injection	3 (37.5)	0	0	0	0	1 (100.0)	1 (20.0)	1 (100.0)
Implant	1 (12.5)	0	0	0	0	0	1 (20.0)	0
Use of external sex hormone that influence reproduction	5 (2.8)	0	0	0	0	1 (9.1)	2 (2.6)	2 (4.9)
Exogenous oestrogen	1 (20)	0	0	0	0	0	0	1 (50.0)
Progestin based to suppress menstruation	4 (80.0)	0	0	0	0	1 (100.0)	2 (100.0)	1 (50.0)
Patients with ovariectomy	3 (1.7)	0	0	0	1 (6.3)	0	2 (2.6)	0
Puberty abnormal	3 (1.7)	0	0	0	1 (6.3)	2 (18.2)	1 (1.3)	0
Male	1 (33.3)	0	0	0	0	1 (50.0)	0	0
Female	2 (66.7)	0	0	0	1 (100.0)	1 (50.0)	1 (100.0)	0
Menstrual cycle disorder (female > 11 years)	22 (20.8)	0	0	0	3 (27.3)	1 (12.5)	5 (11.6)	4 (16.0)
Amenorrhea (female > 11 years)	9 (8.5)	0	0	0	1 (9.1)	0	2 (4.7)	4 (16.0)
Amenorrhea lasted > 3 months	5 (55.6)	0	0	0	0	0	2 (100.0)	2 (50.0)
Other abnormal reproductive condition	3 (1.7)	0	0	0	2 (12.5)	0	1 (1.3)	0
Abnormal hormonal levels								
Thyroid-stimulating hormone	4 (2.2)	0	0	0	1 (6.3)	0	1 (1.3)	2 (4.9)
Follicular-stimulating hormone	1 (0.6)	0	0	0	0	0	0	1 (2.4)
Luteinising hormone	1 (0.6)	0	0	0	0	0	1 (1.3)	0
Oestradiol	1 (0.6)	0	0	0	0	0	1 (1.3)	0
Testosterone	2 (1.1)	0	0	0	0	0	2 (2.6)	0

Of the 179 patients enrolled, 22 of 106 (20.8%) female patients had menstrual cycle disorders. Amenorrhea was reported in nine patients (8.5%) and other abnormal reproductive conditions in three patients (1.7%). In the initial analysis, three patients (1.7%, one male and two females) were reported to have abnormal onset of puberty. However, on further analysis, it was noted that one female child had precocious puberty, which was treated successfully 5 years before starting everolimus treatment. The second patient, a male child, had developed behavioural problems during puberty, which were thought to be secondary to oxcarbazepine and predated everolimus treatment. The third patient, an adult female patient, had abnormal puberty before the start of everolimus too. Thus, abnormal puberty was found to be not related to everolimus treatment.

Abnormal hormone levels of thyroid-stimulating hormone were reported in four patients (2.2%); testosterone levels were abnormal in two patients (1.1%); luteinising hormone, follicular-stimulating hormone, and oestradiol were abnormal in one patient (0.6%) (Table 6).

## Discussion

Based on the understanding of the TSC pathogenesis, the role of everolimus in the management of different TSC manifestations has been extensively evaluated. Studies evaluating everolimus in the treatment of SEGA, angiomyolipoma, and epilepsy have consistently demonstrated its efficacy and tolerability (6–8) which subsequently led to the approval of everolimus in the treatment of these TSC manifestations (14). Studies have also shown that even with prolonged treatment, no new toxicities or complications were observed (6, 10, 11, 13, 20). All these data were obtained from interventional controlled clinical trials. There was a need of real-world evidence on the safety of everolimus. Based on European Medical Agency indications to Novartis (EMA/CHMP/59467/2014, 20 February 2014), the PASS sub-study was performed as part of the TOSCA registry, to evaluate real-world evidence on the safety of everolimus in patients with TSC from 11 European countries. The number of patients recruited in this study varied between participating countries. In addition, as the recruitment was voluntary, the study population does not mirror the prevalent TSC population in each country.

In line with the previously reported everolimus in TSC studies (6–8), the most commonly reported AE was stomatitis, which is effectively managed to minimise the occurrence and severity in TSC patients. The incidence of stomatitis and infections was relatively low in this PASS sub-study (7.8 and 34%) than in the previous EXIST studies (5, 6, 9, 12). Rates of stomatitis in EXIST-1 and EXIST-2 were 31 and 48%, respectively (6, 8). Overall, 55–68% of patients had stomatitis in EXIST-3, which included stomatitis, mouth, aphthous, lip, and tongue ulcerations; mucosal inflammation; and gingival pain (5, 9, 12, 21). The decrease in AEs like stomatitis with longer follow-up could be attributed to better tolerability or better care or both (10). Additionally, the median dose of everolimus was similar to that in the EXIST interventional studies, whereas, the starting doses in the current study were lower, as suggested by 44 patients having their dose increased. In addition, outside of a strict trial protocol, physicians may have been able to interrupt and reduce the dose of everolimus to manage AEs. The rate of infections was reported in about 72% patients in EXIST-1 and 65% of patients in EXIST-2 (8, 11). Similarly, the rate of infections was higher in the EXIST-3 study with nasopharyngitis in 14–23.8%, upper respiratory tract infection in 13–22.4%, and pharyngitis 1–10.2% of the patients (12, 21). However, the incidence of diarrhoea (6.7%) was slightly higher than that reported in the EXIST-3 study (5%) but lower than that of the EXIST-1 and EXIST-2 studies (13% each) (6–8). Notably, PASS sub-study also showed a higher incidence of AEs in children than in the adults (Table 2), as previously reported in the EXIST-1 study (16, 22). It was unknown whether this was due to higher blood levels or increased susceptibility to everolimus.

No new safety signals were observed in the study. Most of the AEs observed in the study were manageable with dose adjustment and/or use of concomitant medication and were of modest severity, with grade 1 or 2 AEs observed in almost 42% of patients. The lower rate of stomatitis or aphthous mouth ulceration and some other AEs in this study compared with that expected from the literature could be due to several reasons including lower starting doses, early interruption or reduction of dose, better education and preparation of patients, or lower median blood concentration of everolimus in TOSCA PASS compared with those of the previous interventional trials (9, 12, 20). In general, there was likely to be a correlation between drug levels and AEs within individual patients as evidenced by the successful treatment of AEs by lowering the patients' dose (Table 4), but it appears that the different individuals have different sensitivities to any particular blood level of everolimus causing AEs. Three deaths were reported during the study. All occurred in adult patients and were deemed by the study investigators as not related to everolimus treatment.

The data on menstrual irregularities concur with the previous findings with respect to clinical features but at lower frequency (9) and confirm that everolimus can cause amenorrhoea and other menstrual irregularities. In most patients, sexual maturation was not affected by everolimus.

In conclusion, the findings from this study are confirmed the manageable safety profile of everolimus in patients with TSC with no new safety signal. The long-term safety data will continue to be collected as per study protocol for the paediatric patients.

## Data Availability Statement

The raw data supporting the conclusions of this article will be made available by the authors, without undue reservation.

## Ethics Statement

The studies involving human participants were reviewed and approved by the study protocol and all amendments were reviewed and approved (if applicable) by independent ethics committee/institutional review board for each centre: National Hospital Organization Central Ethics Committee; Gazi University Clinical Research Ethics Committee; Independent Multidisciplinary Committee on Ethical Review of Clinical Trials; Peking Union Medical College Hospital; Commissie Medische Ethiek UZ Brussel; CNIL (Commission National de l'Informatique et des Libertés), CCTIRS (Comité Consultatif sur le traitement de l'information en matière de recherche dans le domaine de la santé); Comité Etico Investigación Clínica de Euskadi (CEIC-E); Consejeria de Salud y Bienestar Social, Dirección General de Calidad, Investigación, Desarrollo e Innovación, Comité Coordinador de Ética de la Investigación Biomédica de Andalucía; Research Ethics Committee of the University of Tartu (UT REC); Ethikkommission der Medizinischen Universität Graz; North Wales REC–West; Regionala Etikprövningsnämnden i Göteborg; REK–Regionale komiteer for medisinsk og helsefaglig forskningsetikk; Komisja Bioetyczna przy Instytucie Pomnik Centrum Zdrowia Dziecka; Ethikkommission bei der Ludwig-Maximilians-Universitat München; Hokkaido University Hospital Independent clinical research Institutional Ethics Committee; Medical Juntendo University Institutional Ethics Committee; National Center for Chile Health and Deveropment of IRB; Osaka University Hospital of IRB; Ethics Committee at Moscow Institute of Pediatrics and Pediatric Surgery; Peking University First Hospital; Sanbo Brain Hospital Capital Medical University; Tianjin Children's Hospital; Childrens Hospital Of Fudan University; Zhongshan Hospital Fudan University; Fudan University Shanghai Cancer Center; The Second Affiliated Hospital of Guangzhou Medical University; The First Affiliated Hospital, Sun Yan-Sen University; The First Affiliated Hospital Of Guangzhou Medical University; Shenzhen Children's Hospital; West China Hospital, Sichuan University; Xijing Hospital; Children's Hospital of Chongqing Medical University; Wuhan Children's Hospital; The second affiliated hospital of Xi'an jiaotong university; Guangdong 999 brain hospital; Seoul National University Hospital Institutional Review Board; National Taiwan University Hospital (NTUH) Research Ethics Committee (REC); Institutional Review Board of the Taichung Veterans General Hospital; Institutional Review Board of Chung Shan Medical University Hospital; Institutional Review Board, Tungs' Taichung MetroHarbor Hospital; Institutional Review Board of National Cheng Kung University Hospital; Metro South Human Research Ethics Committee; Sydney Children's Hospital Network Human Research Ethics Committee; St Vincents Hospital Human Research Ethics Committee; Royal Melbourne Hospital Human Research Ethics Committee; Siriraj Institutional Review Board; The Institutional Review board, Faculty of Medicine, Chulalongkorn University, 3rd Floor, Ananthamahidol Building, King Chulalongkorn Memorial Hospital; The committee on Human Rights Related to Research Involving Human Subjects; Institutional Review board, Royal Thai Army Medical Department IRB RTA, 5th Floor, Phramongkutklaowejvitya Building, Phramongkutklao College of Medicine; Research Ethics Committee, Faculty of Medicine, Chiang Mai University; Research and Development, Queen Sirikit National Institute of Child Health; Human Research Ethics Committee, Faculty of Health Sciences, University of Cape Town; Shaare Zedek Meidcla center Helsinki comittee; Sheba Medical center Helsinki comittee; Tel Aviv Sourasly Medical center Helsinki comittee; General University Hospital of Patras Ethics Committee; Pendeli Children's Hospital Ethics Committee; General University Hospital of Athens ‘G. Gennimatas’ Ethics Committee; Evaggelismos General Hospital Ethics Committee; General University Hospital of Thessaloniki AHEPA Ethics Committee; General University Hospital of Ionnina Ethics Committee; METC UMC Utrecht; Direcció General de Regulació, Planificació i Recursos Sanitaris; Comité Ético de Investigación Clínica del Hospital Universitario Vall d'Hebron de Barcelona, Generalitat de Catalunya. Departament de Salut; Comité Ético de Investigación Clínica Hospital Universitario La Paz; Dirección General de Ordenación e Inspección, Consejería de Sanidad Comunidad de Madrid, Servicios de Control Farmacéutico y Productos Sanitarios; Comité Etico Investigación Clínica del Hospital Universitario y Politécnico de La Fe; Dirección General de Farmàcia i Productes Sanitaris, Generalitat de Valencia; Comité de Ética de la Investigación de Centro de Granada; Instituto Aragonés de Ciencias de la Salud (IACS); Comité Etico Investigación Clínica Regional del Principado de Asturias; Comité Etico Investigación Clínica Hospital 12 de Octubre; Comité Etico Investigación Clínica Hospital Universitario Virgen de la Arrixaca; Sección de Ordenación e Inspección Farmacéutica Departamento de Salud; Comité Ético de Investigación Clínica del Hospital Universitario del Río Hortega de Valladolid; Comissão de Ética para a Saúde (CES), Centro Hospitalar de Lisboa Ocidental, EPE; Comissão de Ética para a Saúde (CES), Centro Hospitalar do Porto, EPE; Comissão de Ética para a Saúde (CES), Centro Hospitalar Lisboa Central, EPE; Comissão de Ética para a Saúde (CES), Hospital Garcia de Orta, EPE; Comissão de Ética para a Saúde (CES), Centro Hospitalar de São João, EPE; Comissão de Ética para a Saúde (CES), Hospital Professor Doutor Fernando Fonseca, EPE; Comissão de Ética para a Saúde (CES), Centro Hospitalar do Algarve, EPE (Unidade de Faro); LUHS Kaunas Regional Biomedical Research Ethics Committee; Paula Stradina kliniskās universitātes slimnicas, Attīstības biedrības Klīniskās izpētes Ētika Etikas komiteja, Ethics Committee for Clinical Research; Komisija Republike Slovenije za medicinsko etiko; Comitato Etico Indipendente Presso La Fondazione Ptv Policlinico Tor Vergata Di Roma; Comitato Etico Regione Calabria Sezione Centro c/o A.O.U. Mater Domini Di Catanzaro; Comitato Etico Azienda Ospedaliera Universitaria Di Cagliari; Comitato Etico Cardarelli-Santobono c/o Ao Cardarelli; Comitato Etico Per La Sperimentazione Clinica Delle Province Di Verona E Rovigo, Presso Aoui Verona; Eticka Komise Fn Brno; Eticka Komisia Dfnsp Bratislava; Eticka Komisia Pri Dfn Kosice; Eticka Komisia Bratislavskeho Samospravneho Kraja; Comisia Naţională de Bioetică a Medicamentului şi a Dispozitivelor Medicale; Comitato Etico Milano area 1 c/o ASST FBF Sacco-P.O. L. Sacco; Comité de Ética de la Investigación de Centro Hospital Universitario Virgen del Rocío; Comité Ético de Investigación Clínica Fundació Sant Joan de Déu Generalitat de Catalunya. Departament de Salut; Comité Ético de Investigación Clínica Hospital Infantil Universitario Niño Jesús; Consejería de Sanidad Dirección General de Salus Pública Junta de Castilla León; Dirección General de Asistencia Sanitaria, Consejería de Sanidad Gobierno del Principado de Asturias; Dirección General de Planificación, Ordenación Sanitaria y Farmacéutica e Investigación, Consejeria de Sanidad y Política Social Región de Murcia; Ethics Committee at Moscow Institute of Pediatrics and Pediatric Surgery; Paula Stradina kliniskās universitātes slimnicas, Attistibas biedribas Kliniskās izpētes Etikas komiteja, Ethics Committee for Clinical Research; The First Affiliated Hospital of The Fourth Military Medical University; Zhongshan hospital fudan university. Written informed consent to participate in this study was provided by the participants' legal guardian/next of kin.

## Author Contributions

AJ, EB, MB, PC, MD, JF, MF, CH, SJ, JK, JL, AM, RN, VS, MS, RT, and BZ: designing the study, patient accrual, clinical care, data interpretation, and drafting, revising, final review, and approval of the manuscript. PdV, CF, Gd'A, TC, VC, FO'C, JQ, YT, and SY: designing the study, data interpretation, and drafting, revising, final review, and approval of the manuscript. LD'A: designing the study, trial management, data collection, data analysis, data interpretation, and drafting, revising, final review, and approval of the manuscript. RM: designing the study, data analysis, data interpretation, and drafting, revising, final review, and approval of the manuscript. SS: designing the study, trial statistician, data analysis, data interpretation, and drafting, revising, final review, and approval of the manuscript. KB: patient accrual, clinical care, and drafting, revising, final review, and approval of the manuscript. All authors contributed to the article and approved the submitted version.

## Conflict of Interest

JK, EB, TC, VC, PC, Gd'A, JF, PV, MF, CF, CH, SJ, RN, FO'C, JQ, MS, RT, MD, JL, AM, SY, MB, BZ, and AJ received honoraria and support for travel from Novartis. VC received personal fees for consulting, lecture, and travel from Actelion, Bayer, Biogen Idec, Boehringer Ingelheim, Gilead, GSK, MSD, Novartis, Pfizer, Roche, and Sanofi; grants from Actelion, Boehringer Ingelheim, GSK, Pfizer and Roche and personal fees for developing educational material from Boehringer Ingelheim and Roche. PV has been on the study steering group of the EXIST-1, 2 and 3 studies sponsored by Novartis and is a co-PI on two investigator-initiated studies part-funded by Novartis. RN received grant support, paid to her institution, from Eisai and lecture fees from Nutricia, Eisai, Advienne and GW Pharma. YT received personal fees from Novartis for lecture and for copyright of referential figures from the journals and received a grant from the Japanese government for intractable epilepsy research. SJ was partly financed by the EC Seventh Framework Programme (FP7/2007-2013; EPISTOP, grant agreement no. 602391), the Polish Ministerial funds for science (years 2013–2018) for implementation of international co-financed project and the grant EPIMARKER of the Polish National Center for Research and Development No STRATEGMED3/306306/4/2016. JK, PC, CH, JL, and JQ received a research grant from Novartis. KB received grants, personal fees, and non-financial support from Novartis, Alexion, Astellas, BMS, CSL-Henring, Chiesi, Fresenius, Genentech, Hansa, Hexal, MSD, Neovii, Otsuka, Pfizer, Roche, Sandoz, Siemens, Veloxis, Vifor, and Vitaeris, grants from Abbvie, Akebia, Calliditas, CSL Henring, Freseniu, Hookipa, MSD Sharp & Dohme, Quark, Sanofi, Shire, UCB. RM and SS are employees of Novartis, while LD'A was a Novartis employee at the time of manuscript concept approval. This study was funded by Novartis Pharma AG. Novartis has contributed to the study design, data analysis, and the decision to publish. The remaining authors declare that the research was conducted in the absence of any commercial or financial relationships that could be construed as a potential conflict of interest.

## TOSCA Investigators

Japan: Nobuo Shinohara, Shigeo Horie, Masaya Kubota, Jun Tohyama, Katsumi Imai, Mari Kaneda, Hideo Kaneko, Yasushi Uchida, Tomoko Kirino, Shoichi Endo, Yoshikazu Inoue, Katsuhisa Uruno. Turkey: Ayse Serdaroglu, Zuhal Yapici, Banu Anlar, Sakir Altunbasak. Russia: Olga Lvova, Oleg Valeryevich Belyaev, Oleg Agranovich, Elena Vladislavovna Levitina, Yulia Vladimirovna Maksimova, Antonina Karas. China: Yuwu Jiang, Liping Zou, Kaifeng Xu, Yushi Zhang, Guoming Luan, Yuqin Zhang, Yi Wang, Meiling Jin, Dingwei Ye, Weiping Liao, Liemin Zhou, Jie Liu, Jianxiang Liao, Bo YAN, Yanchun Deng, Li Jiang, Zhisheng Liu, Shaoping Huang, Hua Li. Korea: Kijoong Kim. Taiwan: Pei-Lung Chen, Hsiu-Fen Lee, Jeng-Dau Tsai, Ching-Shiang Chi, Chao-Ching Huang. Australia: Kate Riney, Deborah Yates, Patrick Kwan. Thailand: Surachai Likasitwattanakul, Charcrin Nabangchang, Lunliya Thampratankul Krisnachai Chomtho, Kamornwan Katanyuwong, Somjit Sriudomkajorn. South Africa: Jo Wilmshurst. Israel: Reeval Segel, Tal Gilboa, Michal Tzadok, Aviva Fattal-Valevski. Greece: Panagiotis Papathanasopoulos, Antigone Syrigou Papavasiliou, Stylianos Giannakodimos, Stylianos Gatzonis, Evangelos Pavlou, Meropi Tzoufi. Netherlands: A.M.H. Vergeer. Belgium: Marc Dhooghe, Hélène Verhelst, Filip Roelens, Marie Cecile Nassogne, Pierre Defresne, Liesbeth De Waele, Patricia Leroy, Nathalie Demonceau, Benjamin Legros, Patrick Van Bogaert, Berten Ceulemans, Lina Dom. France: Pierre Castelnau, Anne De Saint Martin, Audrey Riquet, Mathieu Milh, Claude Cances, Jean-Michel Pedespan, Dorothee Ville, Agathe Roubertie, Stéphane Auvin, Patrick Berquin, Christian Richelme, Catherine Allaire, Sophie Gueden, Sylvie Nguyen The Tich, Bertrand Godet. Spain: Maria Luz Ruiz Falco Rojas, Jaume Campistol Planas, Antonio Martinez Bermejo, Patricia Smeyers Dura, Susana Roldan Aparicio, Maria Jesus Martinez Gonzalez, Javier Lopez Pison, Manuel Oscar Blanco Barca, Eduardo Lopez Laso, Olga Alonso Luengo, Francisco Javier Aguirre Rodriguez, Ignacio Malaga Dieguez, Ana Camacho Salas, Itxaso Marti Carrera, Eduardo Martinez Salcedo, Maria Eugenia Yoldi Petri, Ramon Cancho Candela. Portugal: Ines da Conceicao Carrilho, Jose Pedro Vieira, José Paulo da Silva Oliveira Monteiro, Miguel Jorge Santos de Oliveira Ferreira Leao, Catarina Sofia Marceano Ribeiro Luis, Carla Pires Mendonca. Lithuania: Milda Endziniene. Latvia: Jurgis Strautmanis. Estonia: Inga Talvik. Italy: Maria Paola Canevini, Antonio Gambardella, Dario Pruna, Salvatore Buono, Elena Fontana, Bernardo Dalla Bernardina. Romania: Carmen Burloiu, Iuliu Stefan Bacos Cosma, Mihaela Adela Vintan, Laura Popescu. Czech Republic: Karel Zitterbart. Slovakia: Jaroslava Payerova, Ladislav Bratsky, Zuzana Zilinska. Austria: Ursula Gruber-Sedlmayr, Matthias Baumann, Edda Haberlandt, Kevin Rostasy, Ekaterina Pataraia. United Kingdom: Frances Elmslie, Clare Ann Johnston, Pamela Crawford. Denmark: Peter Uldall. Sweden: Paul Uvebrant, Olof Rask. Norway: Marit Bjoernvold, Eylert Brodtkorb, Andreas Sloerdahl, Ragnar Solhoff, Martine Sofie Gilje Jaatun. Poland: Marek Mandera, Elzbieta Janina Radzikowska, Mariusz Wysocki. Germany: Michael Fischereder, Gerhard Kurlemann, Bernd Wilken, Adelheid Wiemer-Kruel, Klemens Budde, Klaus Marquard, Markus Knuf, Andreas Hahn, Hans Hartmann, Andreas Merkenschlager, Regina Trollmann.
